# SIMPLEX Enriches Hydrophobic and Lipidated Proteins in Membrane Proteomics Experiments

**DOI:** 10.1002/pmic.70016

**Published:** 2025-08-08

**Authors:** Tingting Li, Alexander Wenger, Cristina Coman, Corinna Borutzki, Michael R. Kreutz, Robert Ahrends

**Affiliations:** ^1^ Department of Analytical Chemistry Faculty of Chemistry University of Vienna Wien Austria; ^2^ Leibniz‐Institut für Analytische Wissenschaften – ISAS – e.V. Dortmund Germany; ^3^ RG Neuroplasticity Leibniz Institute for Neurobiology Magdeburg Germany; ^4^ Leibniz Group ‘Dendritic Organelles and Synaptic Function’ University Medical Center Hamburg‐Eppendorf Center for Molecular Neurobiology ZMNH Hamburg Germany; ^5^ Center for Behavioral Brain Sciences Otto‐von‐Guericke University Magdeburg Germany; ^6^ German Center for Neurodegenerative Diseases (DZNE) Magdeburg Germany

**Keywords:** domains, liquid–liquid extraction, membrane‐enriched sample, synaptic junctions, synaptosomes, transmembrane proteins

## Abstract

Synaptosomes (Syn) and synaptic junctions (SJ) are key neuronal compartments that have been widely characterized in omics studies to understand neurotransmitter‐ and signal transduction‐related events. While synapses are lipid‐rich, multiomics approaches integrating lipids and proteins remain largely underexplored. Liquid–liquid extraction (LLE), commonly used in lipidomics, offers significant potential for multiomics analyses by enabling the extraction of diverse molecular classes from a single sample. However, its impact on protein and phosphoprotein analysis in membrane‐enriched samples has not been thoroughly investigated or compared to one‐phase extraction methods. In this study, we assessed SIMPLEX (Simultaneous Metabolite, Protein, Lipid Extraction), an LLE‐based method, against conventional acetone protein precipitation for mass spectrometry‐based protein identification. SIMPLEX proved superior for proteomics and phosphoproteomics of SJ, achieving a 42% enrichment in membrane proteins compared to acetone precipitation. It enriched not only transmembrane proteins but also S‐palmitoylated proteins. Enriched phosphoproteins included those with beta‐transducin repeats (WD40), Armadillo repeats (ARM), and various transmembrane domains, highlighting the SIMPLEX potential and enhanced performance for multiomics analyses.

## 1 Introduction

Synaptic structure and function are directly related to synaptic protein topology and content [1]. Alterations of the synaptic proteome are associated with several disease states [2, 3] making synaptosome (Syn) and synaptic junction (SJ) preparations widely used means to investigate molecular mechanisms involved in synaptic plasticity as well as neurological disorders [4] such as amyotrophic lateral sclerosis, Alzheimer's disease, and dementia. Syn are sealed organelles enriched in presynaptic compartments, including vesicles, the synaptic cleft, the postsynaptic membrane, and the postsynaptic density [5]. In contrast, SJs are depleted of vesicles following hypoosmotic shock. Compared to whole cell lysates, both Syn and SJ preparations exhibit a unique proteome and enclose high abundance lipids derived from lipid classes such as cholesterol, phosphatidylcholine (PC), phosphatidylethanolamine (PE), phosphatidylserine (PS), and phosphatidylinositol (PI) [6].

Over the past decade, liquid–liquid extraction techniques (LLE) have been at the center of multiomics‐based sample preparation protocols (SIMPLEX [7], MPLEX [8]) as they enable simultaneous access to the samples’ lipidome, metabolome, and proteome [6, 9, 10]. Compared to traditional single‐phase or precipitation methods commonly used in proteomics, the LLE procedure involves additional steps for lipid extraction using organic solvents, such as chloroform or MTBE. For instance, in the SIMPLEX protocol, lipid extraction was first carried out by adding MTBE to solubilize the membrane before the complete protein precipitation started. After inducing phase separation with water, the organic layer was removed. Proteins were then fully precipitated by adjusting the final volume ratio of H_2_O to methanol to 1:4. Although some studies [7, 11] have investigated the impact of LLE and single‐phase methods on protein precipitation, they were mainly focused on total cell lysates or tissue homogenates [7, 11, 12]. To our knowledge, no investigations have assessed the impact of LLE on the proteome characteristics of membrane‐enriched samples. Another limitation of previous studies is the lack of information on the recovery of posttranslational modifications, such as phosphates or fatty acyls.

In this study, we chose the SIMPLEX [7] procedure as a representative LLE and applied it to membrane‐enriched samples—Syn and SJ—to investigate its influence on protein extraction and detection of phosphoproteins in comparison to the classical one‐step acetone precipitation. Additionally, we considered how the SIMPLEX protocol impacts the extraction of transmembrane proteins, accessibility of transmembrane domains, and protein lipidation in order to provide insights that will steer future research into appropriate protein extraction strategies.

## 2 Materials and Methods

### 2.1 Chemicals and Solutions

The ULC/MS‐grade solvents acetonitrile (ACN), Trifluoroacetic acid (TFA), and methanol (MeOH) were bought from Biosolve (Valkenswaard, Netherlands). Formic acid, tert‐butyl methyl ether (MTBE), Tris(2‐carboxyethyl) phosphine hydrochloride (TCEP), Iodoacetamide (IAA), and ammonium bicarbonate were obtained from Sigma‐Aldrich (Hamburg, Germany). Tandem mass tags (TMT) 10plex label reagent was received from Thermo Scientific (Thermo Fisher Scientific, Bremen, Germany). Benzonase Nuclease, Acetone, Tris(hydroxymethyl)aminomethane (Tris‐base), and sodium chloride (NaCl) were purchased from Merck KGaA (Darmstadt, Germany). Mass spectrometry grade trypsin (Trypsin Gold) was purchased from Promega (Madison, WI). Sodium dodecyl sulfate (SDS) was obtained from Roth (Karlsruhe, Germany), tris (hydroxymethyl)‐aminomethane (Tris) from Applichem (Darmstadt, Germany). Phosphatase inhibitor cocktail phosSTOP and Complete EDTA‐free protease inhibitor were bought from Roche Diagnostics. Bicinchoninic acid (BCA) protein assay and TMT 10plex label reagent were acquired from Thermo Scientific. Ammonium bicarbonate (NH_4_HCO_3_), triethylammonium bicarbonate (TEAB), ammonium hydroxide (NH_4_OH), and glycolic acid were supplied by Sigma Aldrich (Hamburg, Germany). All solutions were prepared using ultrapure water (18 MΏ•cm at 25°C).

### 2.2 Animals’ Maintenance

Animals were maintained in the animal facility of the Leibniz Institute for Neurobiology, Magdeburg (Germany) under controlled environmental SPF conditions and group housed on a 12 h light/12 h dark cycle. Food and water were available ad libitum. All animal experimentation was performed following the ARRIVE guidelines for animal experimentation, European Union (EU) regulations, and approved by the local ethical committee. For the preparation of synaptic fractions, four male rats (Han‐Wistar) aged 10–11 weeks were used.

### 2.3 Sample Preparation of Syn and SJ

Syn and SJ isolated from rat hippocampi were prepared by an established subcellular fractionation workflow [13] and checked purification efficiency (Figure S1). Tissue from rat hippocampi was pooled, and samples were homogenized with a manual homogenizer (Potter S). Syn and SJ were collected, washed, and frozen in liquid nitrogen for subsequent analysis. Immunoblot analysis to show enrichment of postsynaptic markers was performed as described previously [14]. Subsequently, all collected biological replicates of Syn and SJ were pooled separately, and technical replicates were generated from the pooled sample.

### 2.4 Protein Extraction by SIMPLEX

SIMPLEX was chosen as a representative of LLE methods to process Syn and SJ samples as described previously [7]. Syn and SJ samples were each processed in three technical replicates. In summary, 225 µL of MeOH was added to the pellet, followed by three freeze‐thaw cycles with intermediate ultrasonication to homogenize the sample. Then, 750 µL of methyl‐tert‐butyl‐ether (MTBE) was added and the samples were incubated for 1 h at 950 rpm and 4°C. A total of 188 µL dd water was further added to induce the phase separation. The upper organic phase was taken out after a 10,000 × *g* centrifugation of 10 min at 4°C, 527 µL methanol was added to the remaining lower phase, and incubated at −20°C for 2 h for protein precipitation. The pellet was collected after a 30 min centrifugation at 13,500 × *g*. The protein pellet of each sample was collected for further use.

### 2.5 Protein Extraction by Acetone Precipitation

Three technical replicates of Syn and SJ were each individually homogenized in a lysis buffer (1% SDS, 150 mM NaCl, 50 mM Tris base, protease inhibitor and phosphatase inhibitor cocktails, pH 7.6) and then centrifuged for 30 min at 4°C at 18,000 × *g*. The supernatant of each sample was transferred into a new clean tube immediately. The proteins were precipitated by adding acetone (v:v, 1:4) and kept in the fridge overnight.

### 2.6 In‐Solution Tryptic Digestion

The proteins of the 12 samples collected from SIMPLEX and acetone precipitation were processed by in‐solution tryptic digestion. The pellet of each sample was resuspended in 8 M urea, 0.1% rapigest (in house‐synthesized), and 50 mM TEAB buffer containing protease inhibitor and phosphatase inhibitor cocktails. The protein concentration was determined by the Pierce BCA Protein Assay kit (Thermo Fisher Scientific, Germany). The same amount of proteins (350 µg) was taken from each sample and volume was normalized by adding TEAB. The proteins were reduced by TCEP (final concentration 10 mM) for 1 h at 22°C and alkylated by IAA (final concentration 40 mM) in the dark at room temperature for 30 min. Then 50 mM TEAB was added to ensure a final concentration of urea lower than 1 M. Proteins were digested for 16 h at 37°C with trypsin (Promega) at an enzyme‐to‐substrate ratio of 1:40 (w/w) using a ThermoMixer (Eppendorf) set at 650 rpm. To terminate the digestion, 100% formic acid was added, reducing the reaction pH below 3. The acidified mixture was then centrifuged at 15,000 × *g* for 10 min. The resulting supernatant was desalted using solid‐phase extraction C18 cartridges (Sep‐Pak, Waters, 50 mg). The peptide concentration was measured using the Nanodrop spectrophotometer (Thermo Fisher Scientific) to calculate amounts. For each sample, 105 µg of peptides was taken out and were subsequently dried under vacuum and stored at −80°C for future use. The experiment was conducted in Protein LoBind microcentrifuge tubes (Eppendorf).

### 2.7 Quality Control

Following digestion, tryptic peptides were subjected to a Monolithic RP‐18 column to assess protein digestion efficiency and reproducibility in sample generation [15]. To ensure consistent performance during measurements, seven in‐house synthesized peptides were measured daily [16]. Additionally, 50 and 500 µg HeLa cells peptide mixtures were measured before and after the experimental set in order to evaluate the performance of the MS platform.

### 2.8 TMT Labelling

The 105 µg dried peptides were reconstituted in 100 mM TEAB buffer (pH 8.5) to a final concentration of 1 µg/µL. A total of 41 µL TMT reagents were added to 105 µg peptides from each replicate and incubated at 25°C in a ThermoMixer for 1 h. The reaction was quenched with 8 µL 5% hydroxylamine and incubated for additional 15 min. A small amount of each sample was pooled and analyzed using a Q‐Exactive HF mass spectrometer to assess labelling efficiency and perform normalization. Equal amounts of all samples were pooled and desalted using C18 cartridges (Sep‐Pak, 50 mg, Waters). The desalted pooled peptides were dried and stored at −80°C for subsequent analysis.

In the labeling scheme, channels 131, 130C, and 130N were assigned to samples prepared using the SIMPLEX method; while channels 129C, 127C, and 126 were assigned to samples prepared using the conventional acetone precipitation method. For the SJ experiment, channels 129N, 130N, and 129C were used for acetone precipitated samples, and channels 127C, 128N, and 128C were designated for samples prepared according to the SIMPLEX procedure. The experiment was carried out in Protein LoBind microcentrifuge tubes (Eppendorf).

### 2.9 Phosphopeptide Enrichment

In total, 560 µg peptides were used for phosphopeptide enrichment TiO_2_ beads (Titansphere, GL Sciences) for phosphopeptides enrichment as previously described [17]. Briefly, the pooled labeled peptides were reconstituted in a freshly prepared loading buffer 1 (80% ACN, 5% TFA, and 1 M glycolic acid) and sequentially subjected to TiO_2_ enrichment at a bead‐to‐peptides ratio of 6:1, 3:1, and 1.5:1. After each enrichment step, the mixture was vortexed, centrifuged, and the beads were pooled. The beads were then washed sequentially with washing buffer 1 (80% ACN, 1% TFA) and washing buffer 2 (10% ACN, 0.1% TFA) to remove nonspecific peptides. Phosphopeptides were eluted using an elution buffer (1% ammonium hydroxide, pH 11.3) and the supernatant was collected and dried.

The resulting pellet underwent a second‐round of enrichment using loading buffer 2 (70% ACN, 2% TFA) and washing buffer 3 (50% ACN, 0.1% TFA). The eluates were acidified; desalted with Oligo R3 (Applied Biosystems) and C18 Disks Empore (3 M), and reconstituted in a hydrophilic interaction liquid chromatography (HILIC) buffer (95% ACN, 0.1% TFA). The phosphopeptides were loaded onto an in‐house packed column (TSKamid gel, 250 µm ID, 15 cm length) and forwarded to fractionation. The experiment was performed using Protein LoBind microcentrifuge tubes (Eppendorf).

### 2.10 Fractionation of Peptides and Phosphopeptides

To reduce sample complexity and enhance proteome coverage, off‐line high pH reversed‐phase fractionation was performed for the non‐phosphorylated peptides on an UltiMate 3000 liquid chromatography (LC) system (Thermo Fischer Scientific, Darmstadt, Germany), while HILIC fractionation was applied to separate the phosphopeptides after TiO_2_ enrichment (UltiMate 3000 LC system). In brief, following TMT labeling, 30 µg of the pooled peptides were loaded onto a Biobasic C18 column and separated with a binary gradient system composed of solvent A (10 mM NH_4_HCO_3_, pH 8.0) and solvent B (10 mM NH_4_HCO_3_, 84% ACN, pH 8.0) ranging from 3% to 50% of solvent B over 65 min. In total, 16 fractions were collected for subsequent peptides analysis.

The enriched phosphopeptides of Syn and SJ were separated using a binary gradient (solvent A: 0.1% TFA, 98% ACN, solvent B: 0.1% TFA) ranging from 15% to 40% of solvent B in 38 min on a polar‐phase TSK gel column, with 10 fractions being manually collected based on peak intensity monitored at 214 nm using UV detection. Phosphopeptide and peptide fractions were dried in a SpeedVac and stored at −80°C until further use. All tubes used in fractionation were LoBind microcentrifuge tubes (Eppendorf).

### 2.11 LC‐MS/MS Measurement

The fractionations of TMT labelled peptides were first reconstituted in 16 µL of 2% ACN, 0.1% TFA buffer. All measurements were performed on a nanoLC system coupled to an Orbitrap mass spectrometer (Dionex UltiMate 3000 RSLCnano, Orbitrap Fusion Lumos, Thermo Scientific). The peptides were first injected and preconcentrated on a C18 Acclaim Pepmap trap column (100 µm × 2 cm; Thermo Fisher Scientific) for 5 min using 0.1% TFA at a flow rate of 20 µL/min. Separation was carried out on a C18 Acclaim Pepmap main column (75 µm × 50 cm; Thermo Fisher Scientific) at 250 nL/min and 60 °C. A binary linear gradient (solvent A: H2O with 0.1% FA; solvent B: 84% ACN 16% H_2_O and 0.1% FA) was applied for peptide separation increasing from 3% to 35% B in 120 min for peptides analysis and from 3% to 42% B in 60 min for phosphopeptide analysis.

For the MS analysis of peptide, mass spectrometer was operated in data‐dependent acquisition mode. Survey scans were acquired at a resolution of 120,000 at m/z 200, covering a mass/charge range of 300–1550 m/z, with an AGC target of 2.0 × 10^5^ and a maximum injection time (IT) of 50 ms. Precursor ions were isolated using a width of 0.4 m/z and fragmented by higher‐energy collision‐induced dissociation (HCD) with a normalized collision energy (nCE%) of 40. The fragment ions were analyzed at a resolution of 60,000 at m/z 200, with an AGC target of 5.0 × 10^4^, an IT of 105 ms, and a Top Speed method with a cycle time of 3 s.

For phosphopeptide analysis, slight modifications were applied: precursor ions were isolated with a width of 0.8 m/z, and the IT for MS/MS scans was increased to 200 ms.

### 2.12 Data Processing

MS raw data were processed using Proteome Discoverer v 2.1 (PD, Thermo Scientific), and searched against the rat protein database, which included both reviewed and unreviewed entries (Uniprot, https://www.uniprot.org, downloaded on 18 January 2018, 36,080 entries) along with a common contaminants database. Mascot and Sequest HT were employed as search engines during the data analysis. A precursor mass tolerance of 10 ppm and a fragment mass tolerance of 0.02 Da were applied. Trypsin was specified as digestion enzyme, allowing for up to two missed cleavages. Full tryptic specificity was required. Carbamidomethylation of cysteine and TMT tags on lysine residues and peptide N‐termini were set as static modifications. Oxidation of methionine was included as a variable modification for all searches, while the phosphopeptide search, also included phosohorylation of serine, tyrosine, threonine residues as variable modifications. The Percolator was employed to evaluate false‐positive rates, and data were filtered based on 1% false‐discovery rate (FDR) at both, peptide‐spectrum match (PSM) and peptide levels. The target‐decoy approach was used to validate FDR for peptide‐spectrum matches.

Phosphorylation site localization was determined using PhosphoRS, embedded within the Proteome Discoverer 2.1, and site probabilities were assessed using ptmRS. Only PSMs with a site localization probability ≥ 90% were retained as confident identifications, and the positions phosphorylation sites within proteins were subsequently determined. Data analysis was performed in R and RStudio, with data visualization achieved using the ggplot2 R package.

### 2.13 Bioinformatics

GO enrichment analysis was performed using the database for annotation, visualization and integrated discovery (DAVID) [18]. The database of synaptic proteins was downloaded from SynGO, an evidence‐based resource for synapse function (https://www.syngoportal.org) [19]. SynGO 1.2 was also utilized to generate visualizations of synaptic function and subcellular localization.

Transmembrane protein analysis was conducted using DeepTMHMM, an online tool for transmembrane helix prediction (https://dtu.biolib.com/DeepTMHMM). GPI‐anchored proteins were identified using NetGPI, a glycosylphosphatidylinositol anchoring prediction tool (https://services.healthtech.dtu.dk/services/NetGPI‐1.1/) [20]. Palmitoylated‐proteins were identified through the S‐palmitoylated protein database, SwissPalm (v2, https://swisspalm.org/) [21] and protein domain analysis was carried out using the SMART database (http://smart.embl‐heidelberg.de/) [22]. The GRAVY (Grand Average of Hydropathy) value of proteins was calculated as previously described by Kyte et al., which involves summing the hydropathy values for each residue and dividing by the length of the protein sequence [23]. The isoelectric point (PI) and molecular weight (Mw) were calculated by R package “peptides” [24].

## 3 Results and Discussion

### 3.1 Rationale for the Research Workflow

Syn and SJ were generated from homogenized hippocampus using sucrose density ultracentrifugation as described previously [13]. The purification efficiency of synaptosomes and synaptic junctions was assessed by western blot (Figure S1). Membrane proteins are hydrophobic and often of low abundance, resulting in low recoveries in proteomics experiments [25]. The results of our initial test, based on a label‐free quantification method with 2 h gradient separation, detected 1203 and 1102 proteins in Syn and SJ preparations, respectively (Table S1). The number of quantified proteins was similar to other studies [26], showing for the Syn fraction replicates a range of 1016–1196 identified proteins. We reasoned that the low identification rate is related to the limitations of the method to access low‐abundant membrane proteins of the synaptic junction proteome. To address this problem, we employed a 2‐D chromatography setup in conjunction with a tandem mass tag (TMT) technique for protein quantification and investigated the impact of SIMPLEX compared to acetone precipitation on protein extraction.

**FIGURE 1:**
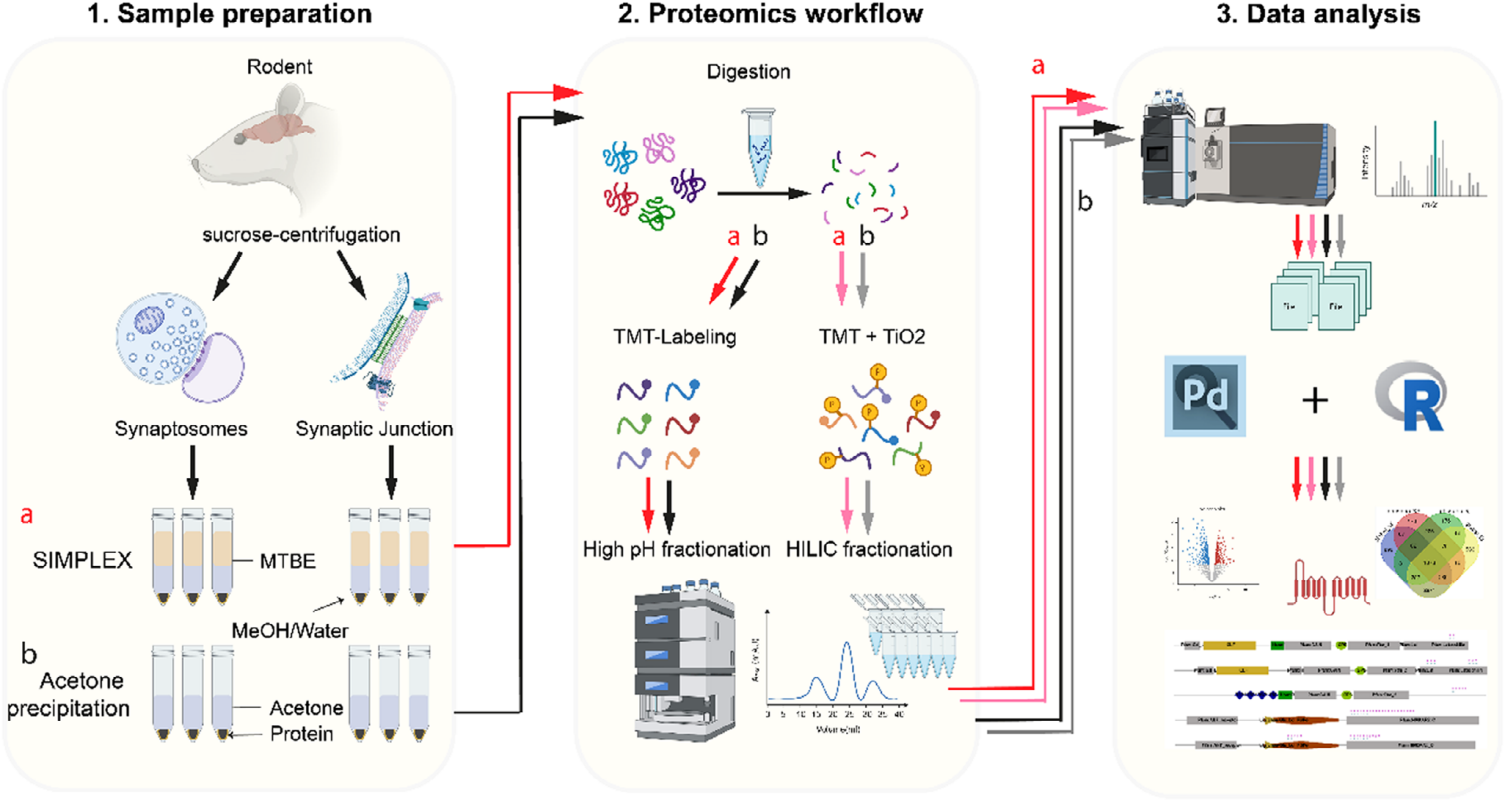
Schematic representation of the integrated proteomic workflow combining SIMPLEX with isobaric chemical labeling and enrichment of post‐translational modifications. The established workflow of Syn and SJ isolation for quantitative comparison of protein extraction efficiencies via an LLE strategy and acetone precipitation strategy. (1) Sample preparation: The Syn and SJ were separated from rat hippocampus via sucrose density ultracentrifugation. Each group contained three technical replicates. SIMPLEX and acetone precipitation methods were used to assess the proteins. The red and light red arrows represent the proteins obtained using the SIMPLEX (a); the black and grey arrows indicate proteins obtained from acetone precipitation (b). (2) Proteomics workflow: In‐solution digestion was conducted. Peptides were collected for TMT labeling and phospho‐enrichment. Further off‐line fractionation was conducted for peptides and phosphopeptides analysis. (3) Data analysis: PD and R were used for data processing after raw data were obtained from MS analysis. All significantly enriched proteins were used for functional annotation.

In brief, ice‐cold MeOH, MTBE, and H_2_O were added to three replicates of each sample type (Syn, SJ), and protein precipitation was performed according to the SIMPLEX protocol. In parallel, protein pellets from acetone precipitation were used for comparison. After in‐solution digestion, 100 µg peptides of each sample were used for TMT labeling and pooling and 30 µg of pooled peptides were taken for protein analysis. The remaining peptides were used for titanium dioxide enrichment for phosphoproteome analysis. To achieve a deep profiling result, 16 offline fractionations of non‐phospho peptides and 8–14 offline fractionations of phosphopeptides were collected for MS measurements (Figure 1).

### 3.2 Workflow Evaluation

**FIGURE 2:**
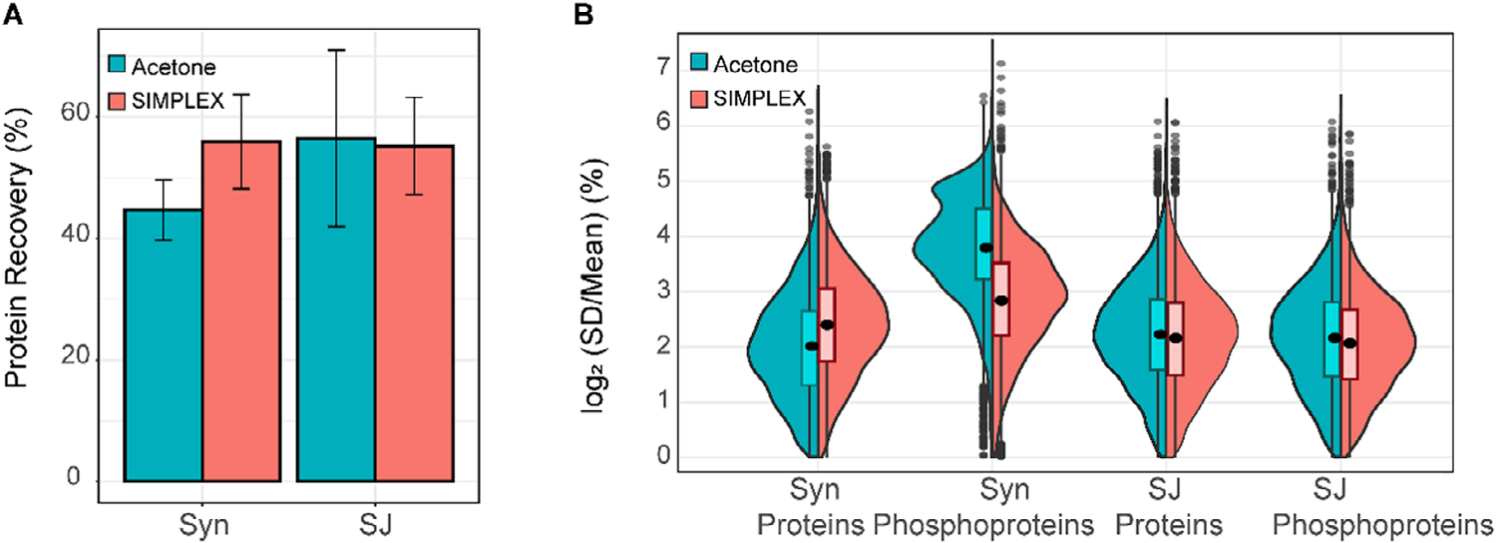
Recovery and evaluation of variability across technical replicates and extractions. (A) Bar plot: protein recovery comparison between SIMPLEX and acetone precipitation for both Syn and SJ. (B) Violin plot: Reproducibility of protein and phosphopeptides data, at the protein level between samples processed by acetone precipitation and SIMPLEX. All values were calculated based on three technical replicates. Log_2_‐transformed coefficient of variation (CV) was generated to plot.

We first investigated the protein recovery for both SIMPLEX and acetone precipitation. The protein recovery ranged from 40% to 71% for acetone precipitation and from 57% to 65% for SIMPLEX, respectively (Figure 2A). The analysis revealed no significant differences in recovery between the two methods for Syn and SJ samples, with comparable average recoveries observed across all sample types. The workflow demonstrated good reproducibility, as indicated by the low median coefficient of variation (CV) within each group. At the protein level, the median CV ranged from 3.71% to 5.15%, while for phosphopeptides, the median CV was slightly higher, ranging from 4.32% to 7.35%. An exception was observed for the acetone precipitation in Syn samples, where the median CV reached 14.24% for phosphopeptides (Figure 2B). Despite this, the variation remained within an acceptable range, supporting the overall reliability of the workflow.

To further evaluate the SIMPLEX workflow, we compared our results with recently published studies [27, 28, 29, 30] on the Syn proteome, which were based on single‐phase extraction methods and isotopic‐labeling. In total, 5687 proteins in Syn and 5,279 proteins in the SJ were quantified, significantly surpassing results of similar studies, which typically report between 3600 and 4800 quantified proteins (Figure S2A). Overall, at the phosphopeptide level, we quantified 7980 phosphopeptides, including 6574 unique phosphosites in Syn, and 9348 phosphopeptides containing 7594 unique phosphosites in SJ. These results are consistent with those of recent studies reporting between 6000 and 9000 unique phosphosites (Figure S2B; Tables S1 and S2). The slightly reduced number of quantified phosphoproteins in our dataset can be attributed to the stricter threshold we applied for site localization probability (≥90%), compared to the 75% threshold used in other studies. Furthermore, the synaptic proteins detected in our study showed slightly higher coverage (25%) in SynGo [19] than the others (16.3%–24.4%) (Figure S2C). All the studies described here had a significant overlap at the protein level, which ranged from 80% to 90% (Figure S3A). However, the relatively low coverage of SynGo is likely rooted in the fact that during the creation of the database, in silico predicted proteins were also included, which are not detectable in any of the workflows described here.

Based on the comparable performance observed with other literature, these findings underscore the robustness of our approach and highlight its compatibility with isotope labeling in proteomics. Furthermore, SIMPLEX offers a specific advantage of enabling the simultaneous extraction of multiple molecular classes. Taken together, these features confirm SIMPLEX as a robust and reliable method, making it an excellent foundation for comprehensive multiomics studies with an excellent proteome coverage for the here analyzed organelles.

### 3.3 SIMPLEX Enriches Larger and More Hydrophobic Membrane Proteins From the Synaptic Junctions

**FIGURE 3:**
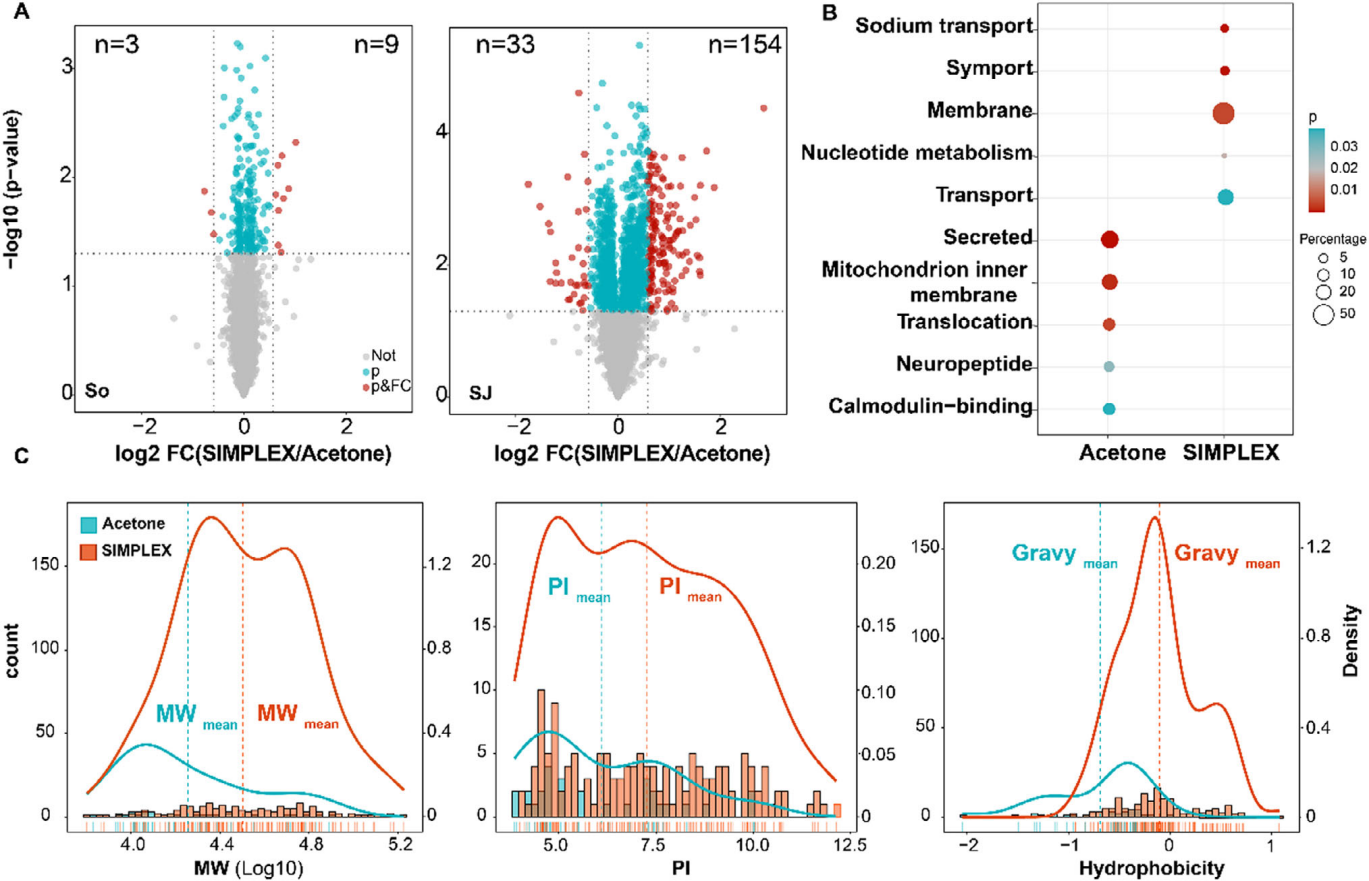
Comparison of proteins extracted from SIMPLEX pellets versus acetone pellets in Syn and SJ. (A) Volcano plots‐based representation of quantified proteins from Syn and SJ samples. Red dots represent proteins that show a fold change > 1.5 or < 0.67, and *p* < 0.05 when comparing SIMPLEX against acetone precipitation, blue dots indicate *p* < 0.05, and grey dots indicate no significant changes in different methods. All values were calculated based on three technical replicates. (B) Functional annotation of the enriched proteins by the two methods in SJ by DAVID. The color represents *p* values (blue: higher *p* values; red: lower *p* values; grey: middle range *p* values), and the sizes of the dots represent the continuous percentage of proteins contributing to the enriched terms, the legend shows representative values for visualization purpose. (C) Comparison of physicochemical properties (molecular weight (MW), isoelectric point (pI), hydrophobicity (GRAVY)) of significantly enriched proteins (shown as the red dots in the volcano plot) of each method. The left y‐axis represents the count of proteins in histogram bars. The right y axis showed overlaid density curve which was plotted by normalized density values showing the distribution of proteins based on physicochemical properties, along with dashed line displays the group median value. The rug plots under the x‐axis show the physicochemical properties values of individual proteins. Colors represent precipitation methods: SIMPLEX (red curve) and acetone precipitation (blue curve).

While the overall protein recovery across Syn and SJ was comparable between SIMPLEX and acetone precipitation, a significant difference was observed in the protein profiles of SJ depending on the precipitation method, whereas Syn protein profiles remained largely unaffected. Specifically, SJ proteins exhibited a strong dependency on the precipitation protocol, whereas Syn proteins consistently fell within a similar range regardless of the method used (Figure 3A). In Syn, the lack of significant differences between SIMPLEX and acetone precipitation corroborates previous findings that multiomics protocols yield results comparable to standard protein methods [8]. Overall, only 4.3% of Syn proteins (245 of 5687 proteins) were differently enriched (*p* < 0.05) when comparing the two methods. Among them, nine proteins in SIMPLEX and three proteins in acetone precipitation showed a fold change greater than 1.5. In contrast, approximately 30% of SJ proteins (1551 of 5279 proteins) showed significant variation (Figure 3A), with 154 proteins (FC > 1.5) in SIMPLEX and 33 proteins (FC > 1.5) in acetone precipitation respectively, emphasizing the strong influence of the precipitation method on the SJ proteome.

Next, differentially enriched proteins in SJ with a fold change (FC) higher than 1.5 were selected for functional annotation (Figure 3B). Proteins enriched in SJ using SIMPLEX were predominantly associated with membranes (57.9%) and transport (23.4%). Notably, proteins associated with symport, which are involved in transport by membrane proteins, were also enriched (5.5%) with the SIMPLEX protocol. In contrast, proteins enriched in SJ following acetone precipitation exhibited different distributions, with a significantly higher presence of secreted proteins (29.6%) and the mitochondrial inner membrane (22.2%). These findings were somewhat unexpected. We had hypothesized that synaptic vesicle proteins in the Syn fraction would be preferentially extracted by the organic phase in SIMPLEX due to their small size and outer lipid bilayer, leading to a notable loss of synaptic vesicle proteins in SIMPLEX. However, the enrichment analysis did not fully align with the expected results. In addition to functional differences, we observed notable variations in the physicochemical properties of the significantly enriched proteins by the two methods. Proteins enriched through SIMPLEX were more hydrophobic compared to those enriched by acetone precipitation (Figure 3C), consistent with the higher proportion of membrane proteins identified in the SIMPLEX‐enriched fractions. The same trend was also observed at the peptides level (Figure S4). Furthermore, proteins extracted by SIMPLEX were larger on average than those extracted by acetone precipitation. Additionally, the isoelectric points (pI) of proteins enriched in SIMPLEX were distributed over a broader range, resulting in a higher average pI value compared to those obtained through acetone precipitation. These differences highlight the unique capabilities of SIMPLEX for enriching specific classes of proteins, particularly hydrophobic and membrane‐associated proteins, in complex proteomes.

### 3.4 SIMPLEX Enriches Membrane and Membrane‐Related Phosphoproteins

**FIGURE 4:**
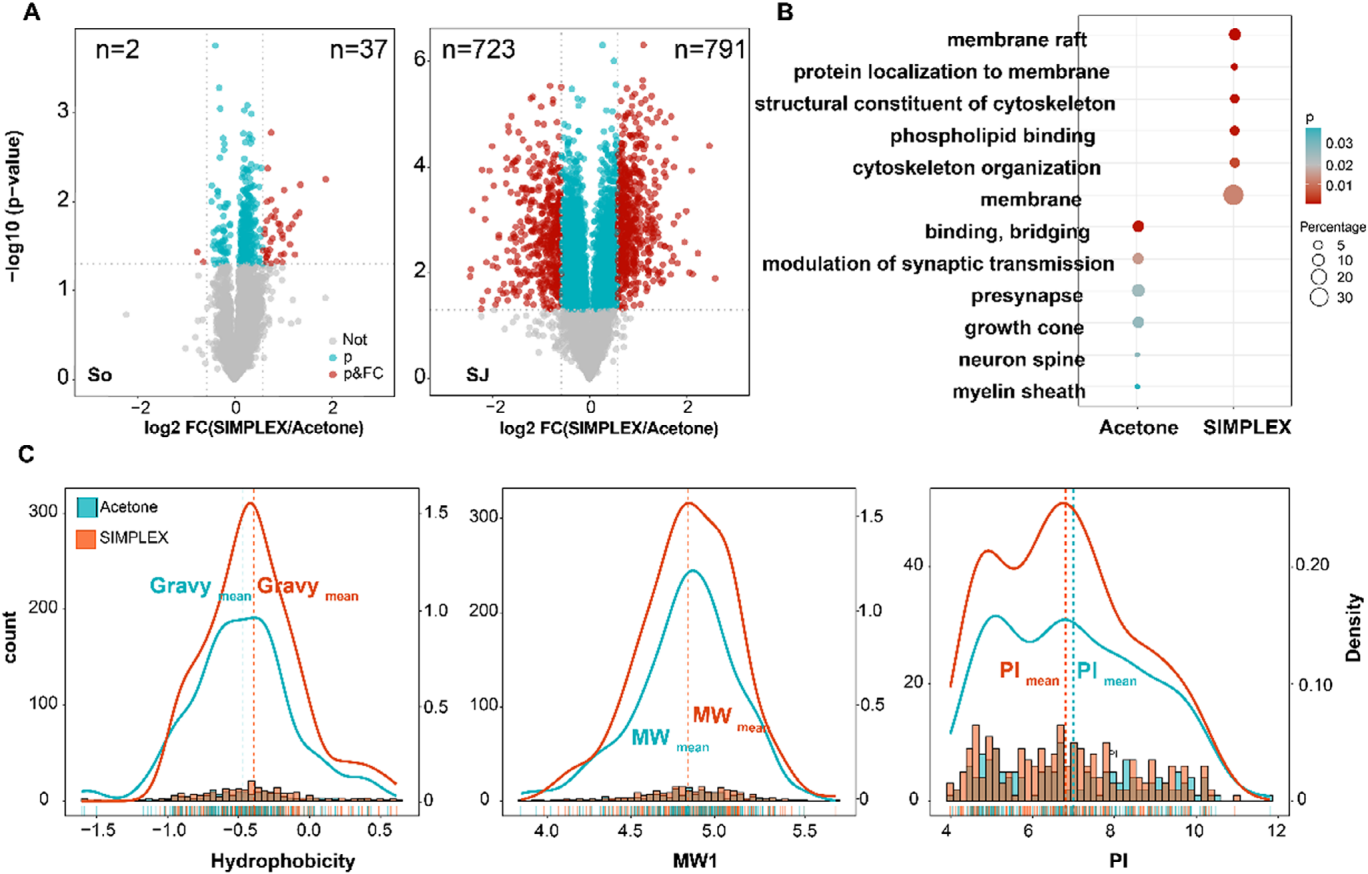
Quantitative analysis of phosphoproteins extracted from SIMPLEX pellets versus acetone pellets in Syn and SJ. (A) Volcano plots‐based representation of quantified phosphor‐peptides in Syn and SJ samples. Red dots represent phosphopeptides that show a fold change > 1.5 or < 0.67, and *p* < 0.05 when comparing SIMPLEX against acetone precipitation; blue dots indicate *p* < 0.05. All values were calculated based on three technical replicates. (B) Functional annotation of the uniquely enriched phosphoproteins (*p* < 0.05, fold change > 1.5 or < 0.67) by the two methods in SJ. The color represents *p* values (blue: higher *p* values; red: lower *p* values; grey: middle range *p* values), and the sizes of the circular encode the continuous percentage of genes contributing to the enriched terms, the legend shows representative values for visualization purpose. (C) Physicochemical properties (MW, pI, hydrophobicity) of the uniquely enriched phosphoproteins (*p* < 0.05, fold change > 1.5 or < 0.67) of SJ in SIMPLEX (red curve) and acetone precipitation (blue curve), respectively. The left y axis represents the count of proteins in histogram bars. The right y axis showed overlaid density curve which was plotted by normalized density values showing the distribution of proteins based on physicochemical properties, along with dashed line displays the group median value. The rug plot under the x‐axis shows the physicochemical properties values of individual proteins. Colors represent precipitation methods: SIMPLEX (red curve) and acetone precipitation (blue curve).

Upon SIMPLEX, a similar result to membrane proteins was achieved for membrane and membrane‐related phosphoproteins. In summary, 360 phosphopeptides derived from 255 phosphoproteins in Syn exhibited differential enrichment (*p* < 0.05) between the two extraction methods (acetone precipitation and SIMPLEX), accounting for 4.5% of the total phosphopeptides. Among these, 39 phosphopeptides had an FC greater than 1.5, with 37 significantly enriched by SIMPLEX and only two enriched with an FC greater than 1.5 in acetone precipitation (Figure 4A). Similar to the protein level, the analysis of the phosphorylated peptides revealed more pronounced differences between the two extraction methods in SJ compared to Syn. In SJ, nearly 51.3% (4794 phosphopeptides from 1029 phosphoproteins) of the total phosphopeptides were differentially enriched (*p* < 0.05). Of these, 563 phosphopeptides from 342 phosphoproteins and 466 phosphopeptides from 277 phosphoproteins, each with a FC greater than 1.5, were enriched using SIMPLEX and acetone precipitation, respectively (Figure 4A).

Although a larger number of phosphopeptides in SJ showed distinct enrichment tendencies between the two methods, the physicochemical properties of the enriched phosphoproteins revealed only minor differences in terms of molecular weight (MW), isoelectric point (pI), and hydrophobicity (Figure 4C). The reason that differences in the physicochemical properties of previously enriched proteins disappeared in the results of subsequent phosphorylation is likely due to the phosphopeptides enrichment procedure itself, which skews the protein distribution, due to the low pH (2–3) of the buffer, TiO_2_ enrichment, and the HILIC fractionation. Most likely the HILIC fractionation has the most effect on this, because larger hydrophobic peptides which are not retained or collected are lost in the first quarter of the separation under high proportion of ACN. This, likely leads to an equilibration of the physicochemical properties, normalizing differences between SIMPLEX and acetone precipitation.

However, functional level differences between the extraction methods remained. Based on GO analysis of the phosphoproteins enriched in SIMPLEX, terms such as “membrane raft,” “structural constituent of the cytoskeleton,” and “cytoskeleton organization” indicate a strong association of these phosphoproteins with membrane structures (Figure 4B). Additionally, terms like “phospholipid binding” and “protein localization to membrane” suggested that SIMPLEX preferentially enriches membrane proteins or proteins with lipid modifications. In contrast, terms enriched using acetone precipitation were broadly distributed among neuronal structural components, from the growth cone and presynapse to neuron spine and myelin sheath. Similar GO annotation patterns were observed in Syn, where phosphoproteins enriched in SIMPLEX were strongly associated with “membrane” (58.6%) and “synaptic vesicle membrane” (24.1%) (Table S3). These findings indicate that SIMPLEX is particularly effective at enriching membrane‐related proteins and phosphoproteins, differentiating it from acetone precipitation, which captures a more diverse set of neuronal structural components.

### 3.5 SIMPLEX Not Only Favors an Enrichment of Membrane Proteins But Also of Proteins With Lipidation

Our results demonstrate that membranes are predominantly enriched at both the protein and phosphoprotein levels in SJ when using SIMPLEX. Unlike acetone precipitation, SIMPLEX incorporates an additional lipid extraction step with MTBE during sample processing. This additional step appears to have a significant impact on SJ, compared to Syn, at both the protein and phosphoprotein levels. Furthermore, membrane proteins showed a stronger tendency for enrichment with SIMPLEX when compared to acetone precipitation. These findings align with comparative studies investigating the effects of SDS lysis buffers, which similarly promote the recovery of membrane‐associated proteins [31].

**FIGURE 5:**
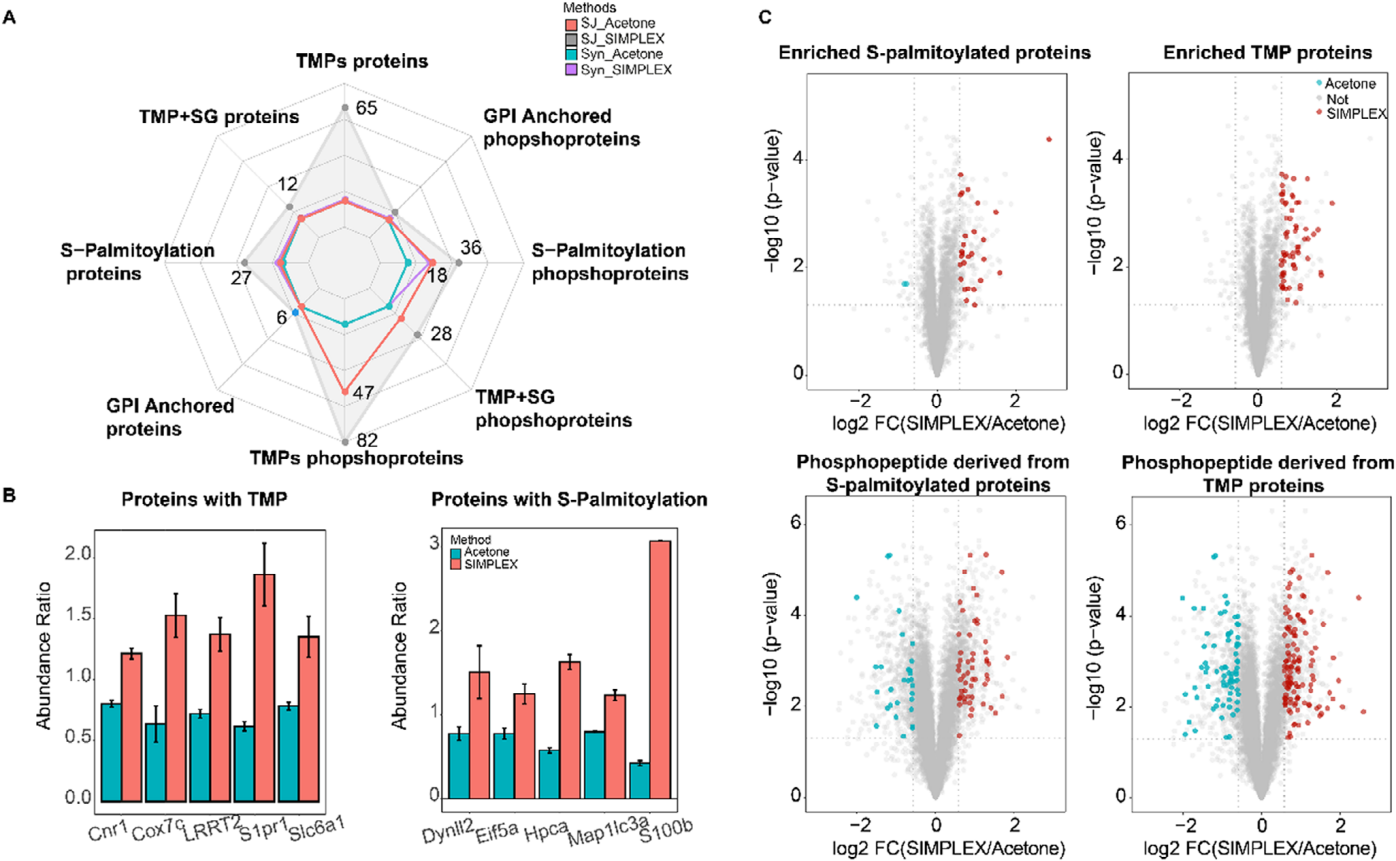
Enrichment analysis of different membrane‐associated proteins and phosphoproteins in Syn and SJ. (A) Radar graph to illustrate the number of significantly enriched transmembrane proteins (TMPs), transmembrane proteins with signal peptides (TMPs+SGs), glycosylphosphatidylinositol‐anchored proteins (GPI‐anchored), and S‐ palmitoylated proteins in Syn and SJ by SIMPLEX and acetone precipitation, respectively. (B) Bar plot showing the abundance ratio of five TMP proteins and five S‐palmitoylated proteins of SJ in different methods. (C) Volcano plot of SIMPLEX versus acetone precipitation. SJ's TMPs and S‐palmitoylated proteins and phosphopeptides were highlighted in different colors. Red dots represent TMPs and S‐palmitoylated proteins/phsophopeptides significantly enriched in SIMPLEX, and blue dots indicate significantly enriched in acetone precipitation. Grey dots showed all proteins/phosphopeptides in the background. All values were calculated based on three technical replicates.

Membranes are composed of lipids forming a phospholipid bilayer and proteins that carry out specialized functions, such as adhesion, signal transduction, and transport [32]. We hypothesized that the lipid extraction step in SIMPLEX disrupts the lipid bilayer, thereby facilitating the extraction of more hydrophobic proteins, including membrane proteins and proteins with lipid modifications such as glycosylphosphatidylinositol (GPI)–anchored proteins and S‐palmitoylated proteins. To test this hypothesis, we performed further enrichment analysis of transmembrane proteins (TMPs), transmembrane proteins with signal peptides (TMPs+SGs), and proteins with lipidation (e.g., GPI‐anchored and S‐palmitoylated proteins) based on the proteomics datasets. The results reveal that SIMPLEX considerably enriches transmembrane proteins (TMPs), transmembrane proteins with signal peptides (TMP+SG), and S‐palmitoylated proteins, as well as their phosphoprotein counterparts, compared to acetone precipitation (Figure 5A). GPI‐anchored proteins and phosphoproteins, while present, show less pronounced differences between the methods.

When comparing differentially enriched proteins derived from S‐palmitoylated and TMP‐associated proteins (Figure 5C), SIMPLEX (highlighted in red) shows a clear dominance (TMPs: 42.2% vs. 0%; S‐palmitolyated: 17.5% vs. 6.3% of enriched proteins) in both categories compared to acetone precipitation. Similarly, the phosphopeptides derived from these protein groups show increased abundance in SIMPLEX (TMPs: 32.1%; S‐palmitolyated: 20.2%) versus acetone precipitation (TMPs: 27.5%; S‐palmitolyated: 14.2%), although the enrichment discrepancy is slightly narrower at the phosphopeptide level. These figures collectively indicate that SIMPLEX is particularly effective in enriching membrane‐associated proteins, such as TMPs and S‐palmitoylated proteins, as well as their phosphoprotein derivatives, when compared to acetone precipitation. This highlights LLE's unique capability to recover hydrophobic proteins, which are essential for studying membrane dynamics and lipid‐associated modifications.

The results of this study indicate that by using SIMPLEX, TMPs are easier to quantify in a complex background. Proteins embedded in synaptic membranes are often expressed at low abundance, limiting their identification or quantification. For instance, S1PR1 (Sphingosine‐1‐Phosphate Receptor 1), the receptor for the bioactive sphingolipid metabolite sphingosine‐1‐phosphate (S1P) crossing the membrane seven times [33], widely involved in many pathological processes such as neuroinflammation and AD, needs more sensitive methods for its extraction and quantification to determine the role of sphingosine such as FTY720 in neurological disorders [34]. In our case, using SIMPLEX, the relative abundance ratio of SIPR1 increased to 1.86 compared to 0.62 with acetone precipitation. Secondly, SIMPLEX also demonstrated significant advantages in increasing the number and abundance of S‐palmitoylated proteins. The abundance of S‐fatty‐acid‐acylated proteins is usually very low, and chemical labeling methods are widely used to improve and detect S‐fatty‐acid‐acylated proteins [35]. By using SIMPLEX on 100 µg starting material, the number of enriched S‐fatty‐acylated proteins reached 27 at the protein level and 36 at the phosphoprotein level (Figure 5C,B). For instance, the neuron‐specific calcium‐binding protein hippocalcin (HPCA), listed in the SwissPalm database, showed a threefold increase in relative abundance with SIMPLEX compared to acetone precipitation, from 0.58 to 1.63 (Figure 5B).

### 3.6 Transmembrane Domains Are Highly Enriched Following SIMPLEX

To investigate the factors contributing to the differences between SIMPLEX and acetone precipitation in the phospho‐enrichment experiment, we conducted a domain analysis of phosphorylated proteins associated with significantly enriched phosphopeptides in the SJ fraction. While the main differences at the protein level were largely driven by transmembrane proteins (TMPs) and S‐palmitoylation, the structural domains of the corresponding proteins revealed more pronounced variations between the two methods.

**FIGURE 6:**
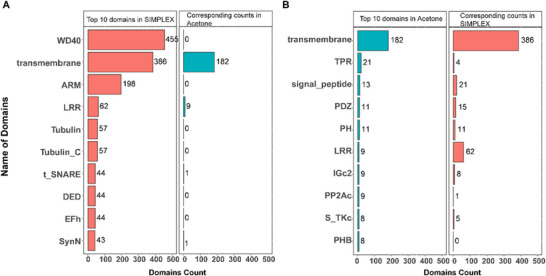
Comparative analysis of enriched domains of phosphoproteins in SIMPLEX and acetone precipitation. (A) The red bars represent the counts of the top 10 most frequent domains in proteins belonging to significantly enriched phosphopeptides in SIMPLEX. In contrast, the blue bars represent the counts of the enriched domains in the acetone precipitation method. (B) The Blue bars represent the counts of the top 10 most frequent domains in proteins belonging to significantly enriched phosphopeptides in the acetone precipitation method. In contrast, the red bars represent the counts of the enriched domains in SIMPLEX.

The analysis showed that the transmembrane domain was the only structural domain prominently enriched by acetone precipitation, with a count of 182. In contrast, SIMPLEX enriched a broader range of functional domains, including beta ‐ transducin repeats (WD40), transmembrane, and armadillo repeats (ARM) domains, with counts ranging from 198 to 455, as shown in Figure 6A. Other domains enriched by SIMPLEX, such as LRR (leucine‐rich repeat), Tubulin, and t‐SNARE, ranged from 43 to 62, representing substantial diversity in functional categories of SIMPLEX. Conversely, acetone precipitation enriched far fewer domains, with most counts ranging from 8 to 21 (Figures 6B and S5). This observation was further confirmed by the protein domain analysis at species level. In total, 101 domain species were enriched in SIMPLEX, including 60 unique protein domain species not found in acetone precipitation. While, acetone precipitation contained 38 unique domain species, which is only half the number of SIMPLEX, representing substantial increase in diversity in functional membrane categories using SIMPLEX (Figure S6).

Of note, SIMPLEX was particularly effective in enriching domains critical for neuronal protein function. For instance, the WD40 domain, known for its role in synaptic vesicle binding via the leucine‐rich repeat kinase 2 (LRRK2) gene [32], was enriched 455 times in SIMPLEX but was not detected in acetone precipitation. Similarly, the LRR domain, which is important for neural activity, was enriched 62 times in SIMPLEX compared to just nine times in acetone precipitation, reflecting a sevenfold increase.

These findings underscore SIMPLEX's superior ability to enrich structural and functional domains associated with neural processes. This enhanced enrichment suggests that SIMPLEX is more effective in recovering phosphoproteins with critical roles in membrane dynamics and neuronal function, providing a more comprehensive view of the phosphoproteome compared to acetone precipitation. This makes SIMPLEX particularly valuable for studies focusing on membrane‐associated proteins and phosphoproteins in synaptic contexts.

## 4 Conclusion

Our study demonstrates the effectiveness of the SIMPLEX protocol when combined with organelle purification and TMT for analyzing the SJ proteome and phosphoproteome, surpassing traditional one‐phase protein precipitation method. By combining SIMPLEX, known for its ability to generate protein, lipid, and metabolite fractions for multiomics studies, with the TMT labeling, we not only achieved an increased detection of proteins compared to the label‐free strategy but also enhanced enrichment of membrane‐associated and lipidated proteins relative to acetone precipitation. This makes it particularly valuable for studying synapse‐related processes, such as neuronal plasticity and lipid signaling, where proteins and lipids are intricately linked.

Our findings revealed that SIMPLEX is highly effective at enriching hydrophobic and membrane‐bound proteins, as well as their phosphoprotein counterparts, especially in the lipid‐rich SJ fraction. The method's ability to preserve protein modifications and capture key structural domains, such as WD40, ARM, and LRR, underscores its utility in unraveling the molecular complexity of the synaptic structure. Due to the fact that hydrophobic peptides or proteins showed poor retention on the HILIC materials under high proportion of organic solvent, we expect that the combination of SIMPLEX and SP4 (solvent precipitation, single ‐pot, solid ‐ phase ‐ enhanced sample preparation) [36]–a optimized SP3 (single ‐ pot, solid ‐ phase ‐ enhanced sample preparation) method using inert glass beads‐would further enhance the recovery of hydrophobic proteins.

One limitation of this study is that our data analysis relied mainly on enrichment analyses and in silico prediction tools, which may have resulted in the mis‐annotations of some proteins. However, our study illustrates the preferential extraction trend of SIMPLEX. It is also important to note that the membrane associations states of proteins such as peripheral or integral cannot be differentiated by MS data alone.

In conclusion, SIMPLEX offers a robust and reproducible workflow for proteomics and phosphoproteomic studies, enabling the deeper profiling of synaptic proteomes and phosphoproteomes. Its compatibility with advanced fractionation and labeling techniques establishes it as a valuable tool for multi‐omics studies, particularly in neuroscience, where, besides the proteome, detailed insights into the lipidome and metabolome are essential for understanding synaptic biology.

## Conflicts of Interest

The authors declare no conflicts of interest.

## Supporting information




**Supporting File 1**: pmic70016‐sup‐0001‐SuppMat.docx

**Supporting File 2**: pmic70016‐sup‐0002‐SuppMat.xls

## Data Availability

The mass spectrometry proteomics data have been deposited to the ProteomeXchange Consortium via the PRIDE partner repository with the dataset identifier PXD058138.
