# Supplementary material for: SIMPLEX Enriches Hydrophobic and Lipidated Proteins in Membrane Proteomics Experiments
Source: Proteomics. 2025 Aug 8;25(16):28–39. doi: 10.1002/pmic.70016 (PMC12381908; doi:10.1002/pmic.70016)
Supplement: Supplementary file 1 — Supporting File 1: pmic70016‐sup‐0001‐SuppMat.docx [file PMIC-25--s002.docx]

Supplementary Data

| Study | Study | Sample Type | Precipitation Method | Enzyme | Quantitative Strategy | Fractionation |
| --- | --- | --- | --- | --- | --- | --- |
| Silbern et al. | Silbern | Rat synaptosomes | Methanol/chloroform | Trypsin | TMT | 12 fractions |
| Eagelson et al. | Eagelson | Mice synaptosomes | Acetone | --- | iTRAQ | 11 fractions |
| Kong et al. | Kong | Rat synaptosomes | Acetone | LysC+Trypsin | TMT | 12 fractions |
| This study | Our study | Rat synaptic junction/  synaptosomes | Acetone | Trypsin | TMT | 16 fractions |

Table S1: The key experimental setups used in the referenced literature for peptide analysis in the proteome experiment.

| Study | Study | Sample Type | Precipitation Method | Enzyme | Quantitative Strategy | Fractionation | Erichments |
| --- | --- | --- | --- | --- | --- | --- | --- |
| Silbern et al. | Silbern | Rat synaptosomes | Methanol/chloroform | Trypsin | TMT | 24 fractions | TiO2 |
| Keller et al. | Keller | Rat synaptosomes | Methanol/chloroform | LysC+Trypsin | Dimethlation | 10 fractions | TiO2 |
| This study | Our study | Rat synaptic junction/  synaptosomes | Acetone | Trypsin | TMT | 8-14 fractions | TiO2 |

Table S2: The key experimental setups used in the referenced literature and in our studies in the phosphopeptide analysis within the phospho-proteome experiment.


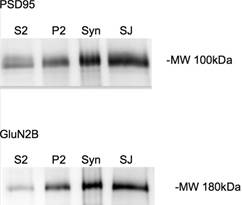


**Figure S1: Purification efficiency of synaptosomes and synaptic junctions.**Western blot analysis after subcellular fractionation of rat forebrain protein preparations. Enrichment of the postsynaptic scaffold PSD95 and the GluN2B subunit of NMDAR is clearly visible in synaptosomes and synaptic junctions’ fractions from rat hippocampus as described in Borgmeyer and Coman et al. 10 µg of protein from supernatant (S2), heavy membrane pellet 2 (P2), synaptosomes (Syn) and synaptic junctions (SJ) fractions were loaded on SDS-page for subsequent immunoblot analysis.


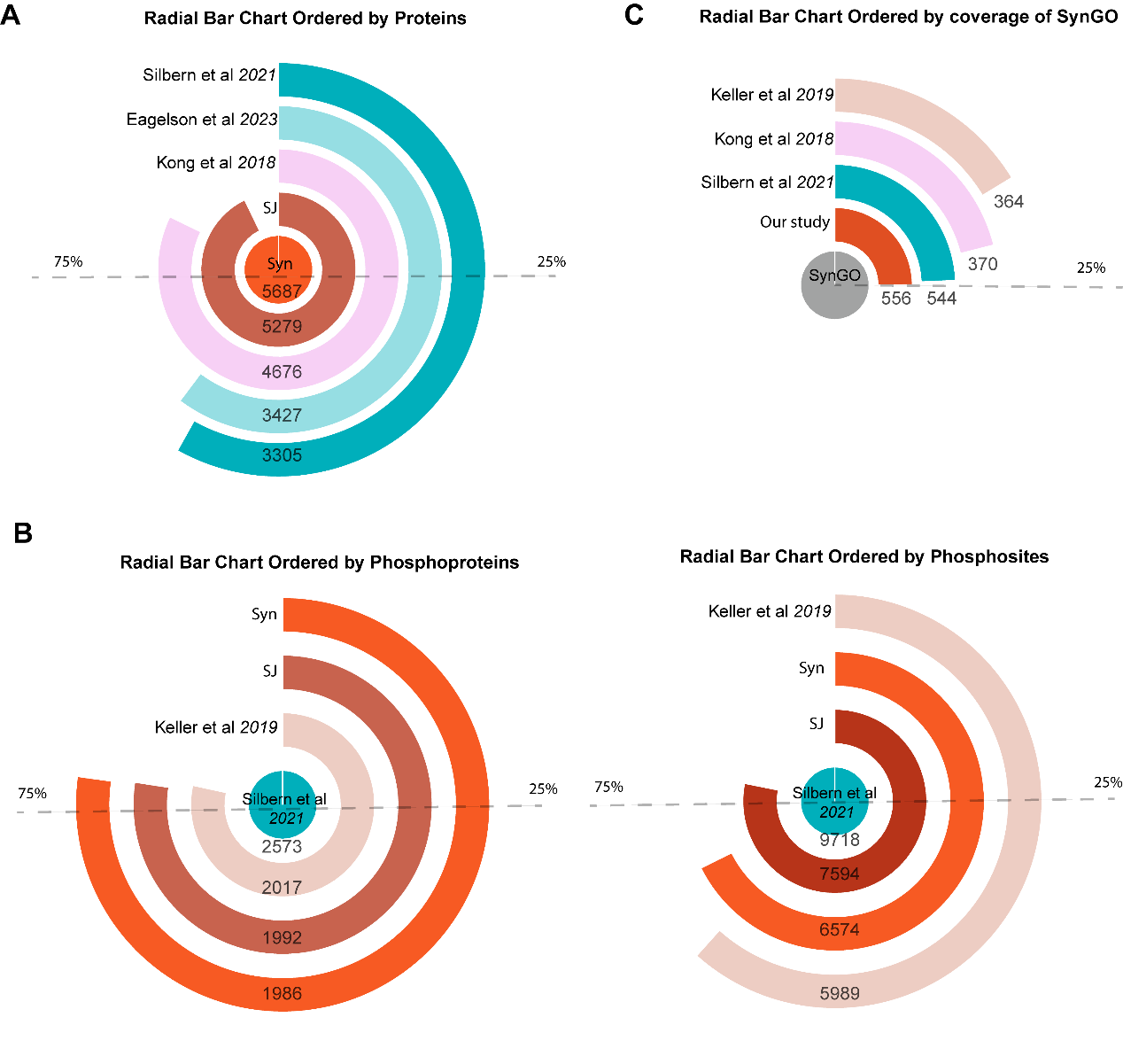


**Figure. S2 Evaluation of proteomics and phosphoproteomics results.** Comparison of the SIMPLEX workflow with recent literature in radical bar charts: The color of the bars represents different studies. In each chart, the largest number of identifications is assigned to the center; the percentage indicates the proportion of obtained results relative to the inner circle. A larger angular span indicates a smaller value. Radial Bar Charts showed the comparison results of proteins (A), phosphoproteins, and phosphosites (B) between our study and recent synaptic proteome literature Eagelson et al.^[29]^, Kong et al.^[28]^, Sibern et al.^[27]^, Keller et al.^[30]^, and coverage of the SynGO database (C).


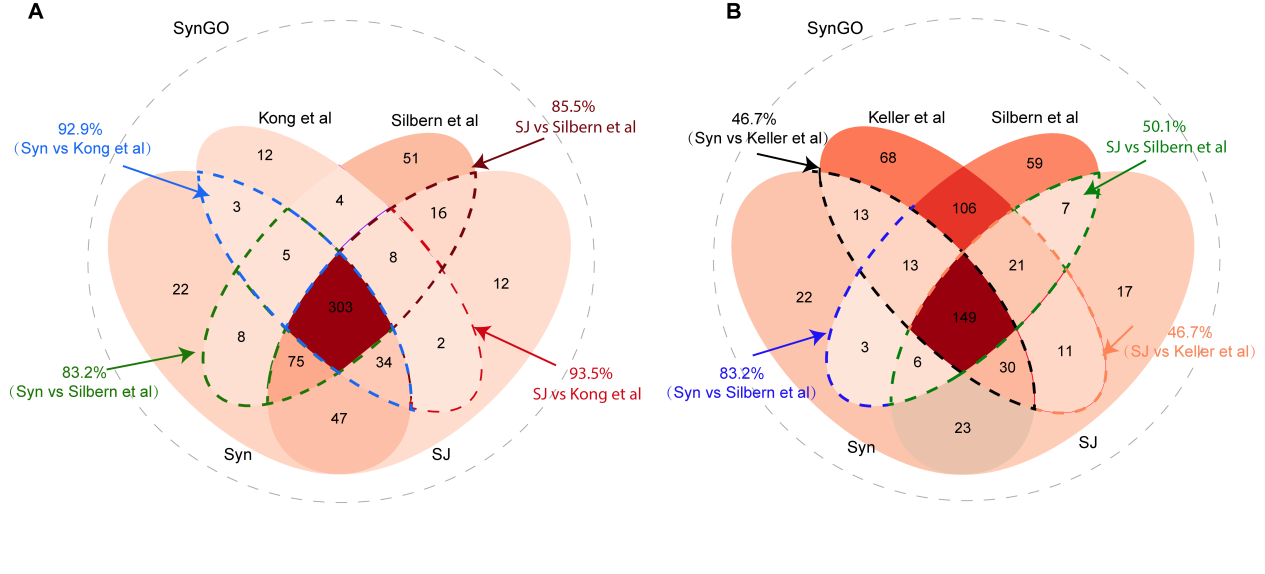


**Figure S3: Comparative analysis of SynGO protein coverage in our study and other literature.** The comparsison of coverage of our workflow with SynGO database to that of the recent literature with SynGO. The comparison results of proteins (A) and phospho-proteins (B) between our study and recent synaptic proteome literature. Venn diagram displaying the overlap of proteins and phospho-proteins detected in our study and recent synaptic proteome studies, based on the SynGO database coverage.The overlap of synaptic proteins in this study and in other studies, ranging from 80% to 90% at protein level and 46.7% to 51.3% at the phosphoprotein levels.

| GO_Term | Type | Condition | Percentage | p |
| --- | --- | --- | --- | --- |
| SNARE complex assembly | Syn_phospho-proteins | SIMPLEX | 10.3 | 4.50E-03 |
| vesicle fusion | Syn_phospho-proteins | SIMPLEX | 10.3 | 9.40E-03 |
| positive regulation of calcium ion-dependent exocytosis | Syn_phospho-proteins | SIMPLEX | 10.3 | 9.40E-03 |
| calcium ion regulated exocytosis | Syn_phospho-proteins | SIMPLEX | 10.3 | 2.70E-02 |
| vesicle-mediated transport | Syn_phospho-proteins | SIMPLEX | 13.8 | 4.30E-02 |
| synaptic vesicle membrane | Syn_phospho-proteins | SIMPLEX | 24.1 | 3.50E-04 |
| membrane | Syn_phospho-proteins | SIMPLEX | 58.6 | 1.40E-03 |
| SNARE complex | Syn_phospho-proteins | SIMPLEX | 13.8 | 0.0032 |
| synaptic vesicle | Syn_phospho-proteins | SIMPLEX | 20.7 | 0.0064 |
| axon | Syn_phospho-proteins | SIMPLEX | 27.6 | 0.017 |
| presynapse | Syn_phospho-proteins | SIMPLEX | 20.7 | 0.029 |
| SNAP receptor activity | Syn_phospho-proteins | SIMPLEX | 13.8 | 0.00078 |
| SNARE binding | Syn_phospho-proteins | SIMPLEX | 17.2 | 0.0011 |
| macromolecular complex binding | Syn_phospho-proteins | SIMPLEX | 24.1 | 0.016 |
| protein binding | Syn_phospho-proteins | SIMPLEX | 48.3 | 0.023 |

**Table S3:** Function annotations of unique enriched phospho-proteins of SIMPLEX in Syn by DAVID.


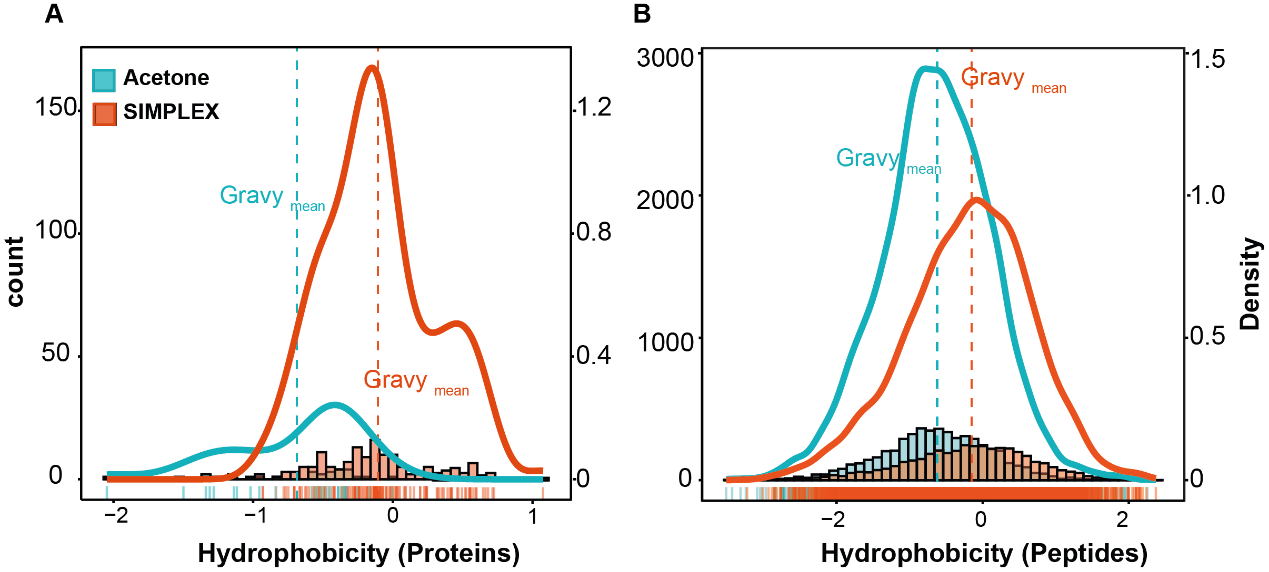


**Figure S4.** Hydrophobicity of the uniquely enriched proteins and peptides (p < 0.05, fold change > 1.5 or < 0.67) of SJ in SIMPLEX (red curve) and acetone precipitation (blue curve), respectively. The left y axis represents the count of proteins in histogram bars. The right y axis showed overlaid density curve which was plotted by normalized density values showing the distribution of proteins based on physicochemical properties, along with dashed line displays the group median value. The rug plot under the x-axis shows the physicochemical properties values of individual proteins. Colors represent precipitation methods: SIMPLEX (red curve) and acetone precipitation (blue curve). Figure A shows the hydrophobicity (GRAVY) of significantly enriched proteins in each method; figure B shows the hydrophobicity comparison of the two methods at the peptide level.


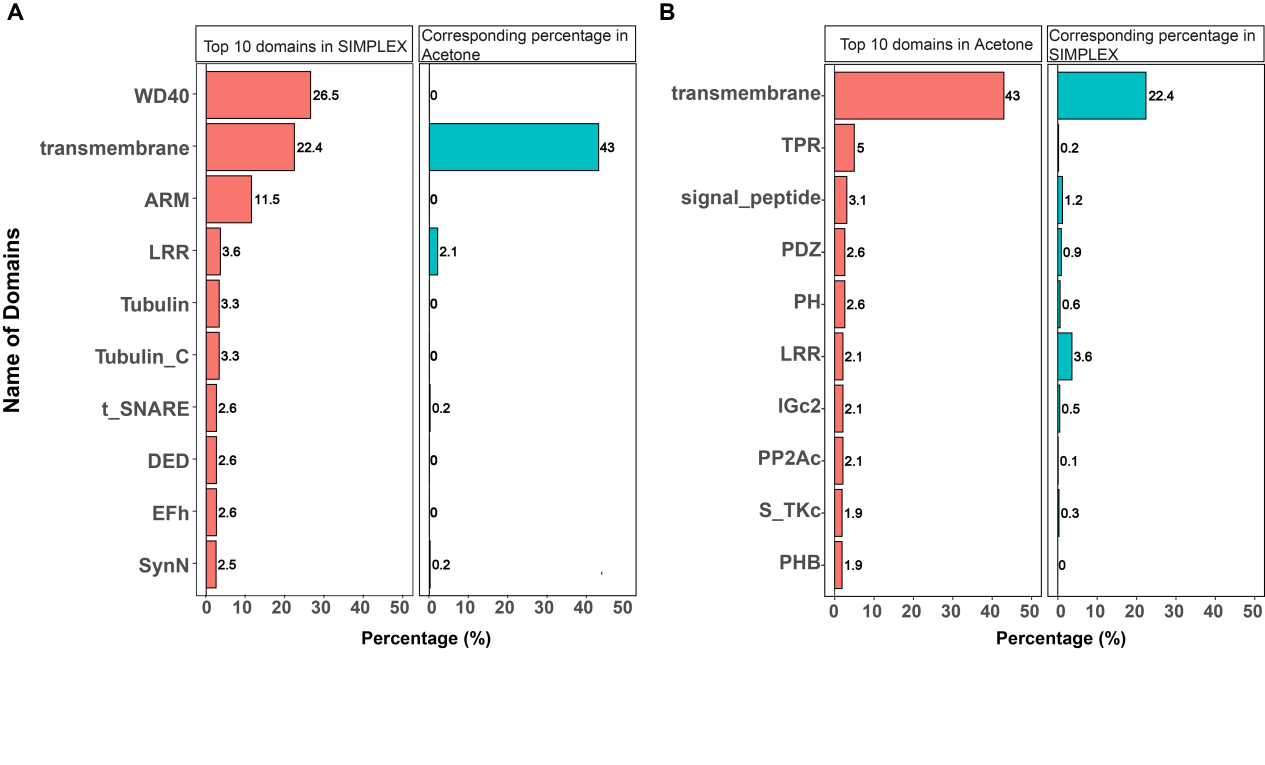


**Figure S5. Comparative analysis of enriched domains of phosphoproteins in SIMPLEX and acetone precipitation.** (A) The red bars represent the relative percentage of the top 10 most frequent domains identified in SIMPLEX. The blue bars represent the relative percentage of the same domains in the acetone precipitation method. (B) The blue bars indicated the relative percentage of the top 10 most frequent domains identified in the acetone precipitation method with the corresponding values (red bars) in SIMPLEX. The percentage was calculated as the proportion of top 10 domains relative to the total number of enriched domains in the respective method. The analysis revealed that the transmembrane domain was the only structural domain prominently enriched by acetone precipitation, accounting for 43% (182 counts) of all enriched domains in the acetone precipitation. In contrast, SIMPLEX enriched a broader range of functional membrane domains, including WD40 (26.5%, 455 counts), transmembrane (22.4%, 366 counts), and ARM domains (11.5%, 198 counts)


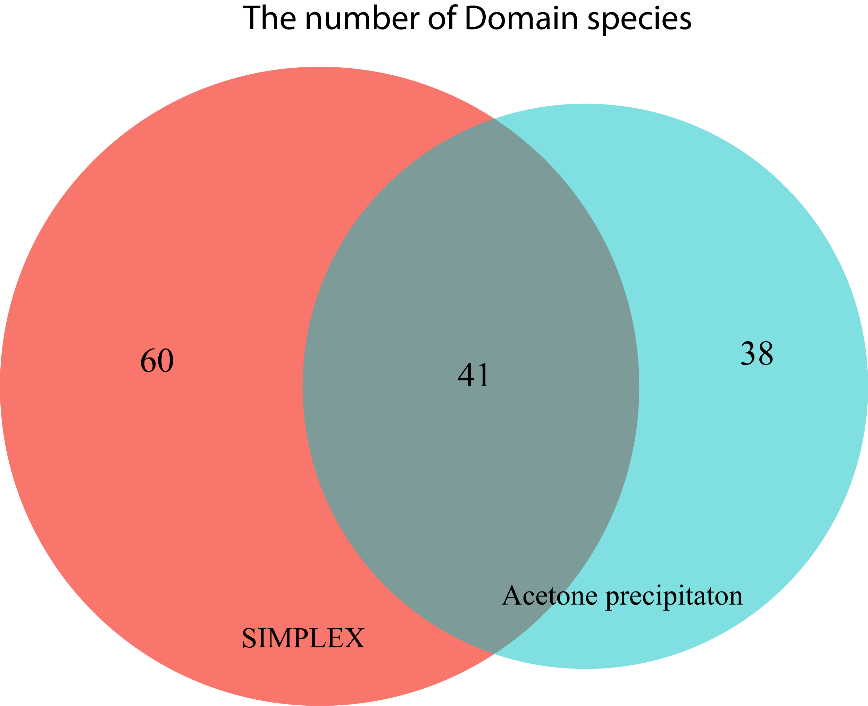


**Figure S6: The Venn plot illustrated the comparison of domain species enriched in SIMPLEX and acetone precipitation.** Red: SIMPLEX; Blue, acetone precipitation. In total, 101 domain species were enriched in SIMPLEX, including 60 unique domain species not found in acetone precipitation. While acetone precipitation enriched 41 species shared between the two methods and 38 unique domain species, which is only half the number of SIMPLEX, representing substantial increase in diversity in functional membrane categories using SIMPLEX.
